# Tumor Angiogenesis

**DOI:** 10.1155/2010/761671

**Published:** 2010-09-19

**Authors:** Arkadiusz Dudek, Kalpna Gupta, Sundaram Ramakrishnan, Debabrata Mukhopadhyay

**Affiliations:** ^1^Division of Hematology, Oncology and Transplantation, Department of Medicine, University of Minnesota, Minneapolis, MN, USA; ^2^Department of Pharmacology, University of Minnesota, Minneapolis, MN, USA; ^3^Department of Biochemistry and Molecular Biology, Tumor Angiogenesis, Vascular Biology and Nanotechnology Laboratory, Mayo Clinic Foundation, Rochester, MN, USA

In a seminal *New England Journal of Medicine* article, published 40 years ago, Folkman proposed the concept that angiogenesis could be a target for cancer therapy [1]. This proposal emerged from early observations that some tumors cells were capable of stimulating endothelial cells to form new capillary sprouts and that nonvascularized tumors were held in a dormant state, unable to grow beyond a size larger than 2-3 mm^3^. These and related observations led Folkman to cautiously conclude that “…the mechanism by which tumor implants stimulate neovascularization must be well understood before therapy based upon interference with angiogenesis can be devised.” Since then, a wide variety of antiangiogenic therapies have been tested in clinical trails, with relatively modest improvements in patient outcomes and unforeseen therapeutic challenges. These initial setbacks call for a reinvigorated research effort to better understand the complex molecular and biological relations between tumor cells and endothelial cells within a neoplasm and the development of new, more effective, therapeutic tools targeting tumor angiogenesis. Encouraging this research is the theme of this special issue of the *Journal of Oncology*.

Folkman's original proposal to target angiogenesis for cancer therapy relied on several assumptions. One key assumption was that solid tumors would only grow beyond a size of 2-3 mm^3^ after vascularization was established, leading to more efficient diffusion of oxygen, nutrients, and wastes. A second assumption was that tumor cells produce angiogenesis in part by stimulating the growth of endothelial cells from surrounding vessels. A third assumption was that blocking angiogenesis would suppress tumor growth and result in resumption of tumor dormancy. The fourth assumption was that antiangiogenic cancer therapy could be delivered chronically because angiogenic activity is of minimal importance to healthy tissues. 

Since Folkman's landmark paper, several factors have been recognized as critical for the induction of tumor angiogenesis, with one of these being vascular endothelial growth factor (VEGF) and its interaction with VEGF receptors. Many therapeutic strategies were developed to either block VEGF, block VEGF binding to its receptor, or interfere with intracellular signaling in the VEGF receptor pathway. Early work in preclinical models led to clinical studies and the development of a multitude of antibodies and small molecules that target tumor angiogenesis. Several clinical trials provided encouraging evidence supporting the use of these agents in the treatment of breast cancer, lung cancer, kidney cancer, and colon cancer. However, resistance to antiangiogenic agents was seen in clinical trials, challenging the notion that endothelial cells supplying the tumor vasculature are genetically stable and, therefore, unlikely to develop mutations that lead to such resistance. Furthermore, there are indications that the initial response to clinical antiangiogenic agents may lead to the development of more aggressive tumors. Since then we have identified several other pathways involved in the biology of angiogenesis and also identified several mechanisms leading to the resistance. 

Lymphangiogenesis and lymph node metastases are another critical determinant of tumor progression and may even be responsible for the emergence of resistance to cancer therapy. Yu and colleagues and Kitadai review the literature pertaining to lymphangiogenesis in breast and gastric cancers, respectively. Though endothelial in origin, lymphatic endothelial cells are distinct with specific cell surface receptors. Their origin, proliferation, and survival may pose a major challenge in treating cancer with angiogenesis-based therapy. Therefore, therapies targeting lymphangiogenesis, in addition to angiogenesis, offer promising prospects to achieve improved cancer management.

This special issue set out to address several issues related to tumor angiogenesis, such as, the mechanisms by which tumor cells acquire the capability to “turn on” the angiogenic switch, the influence of the tumor microenvironment on angiogenesis (Campbell et al., Schmidt and Varner, Tilan and Kitlinska, Rigogliuso et al., Oettgen), types of resistance to antiangiogenic therapy (Grepin and Pages, Rahman et al.), and the development of novel angiogenic strategies (Colema et al., Corti et al., Otten, Bokemeyer, and Fiedler, Seyfried and Mukherjee). Invited papers address several of the above topics and this issue is divided into the following chapter subgroups: biology of tumor angiogenesis, discovery of new angiogenesis targets for cancer therapy, disease-specific tumor angiogenesis, and disease-specific cancer therapies. There will be several review and research papers addressing all those issues.

*Arkadiusz Dudek* *Arkadiusz Dudek* *Kalpna Gupta* *Kalpna Gupta* *Sundaram Ramakrishnan* *Sundaram Ramakrishnan* *Debabrata Mukhopadhyay* *Debabrata Mukhopadhyay*
